# Perspective: the evolution of hormones and person perception—a quantitative genetic framework

**DOI:** 10.3389/fpsyg.2024.1395974

**Published:** 2024-06-17

**Authors:** Christopher I. Gurguis, Tyler S. Kimm, Teresa A. Pigott

**Affiliations:** Department of Psychiatry and Behavioral Sciences, McGovern Medical School at UTHealth, Houston, TX, United States

**Keywords:** evolutionary psychiatry, hormones, quantitative genetics, person perception, evolutionary theory

## Abstract

Evolutionary biology provides a unifying theory for testing hypotheses about the relationship between hormones and person perception. Person perception usually receives attention from the perspective of sexual selection. However, because person perception is one trait in a suite regulated by hormones, univariate approaches are insufficient. In this Perspectives article, quantitative genetics is presented as an important but underutilized framework for testing evolutionary hypotheses within this literature. We note tacit assumptions within the current literature on psychiatric genetics, which imperil the interpretation of findings thus far. As regulators of a diverse manifold of traits, hormones mediate tradeoffs among an array of functions. Hormonal pleiotropy also provides the basis of correlational selection, a process whereby selection on one trait in a hormone-mediated suite generates selection on the others. This architecture provides the basis for conflicts between sexual and natural selection within hormone-mediated suites. Due to its role in person perception, psychiatric disorders, and reproductive physiology, the sex hormone estrogen is highlighted as an exemplar here. The implications of this framework for the evolution of person perception are discussed. Empirical quantification of selection on traits within hormone-mediated suites remains an important gap in this literature with great potential to illuminate the fundamental nature of psychiatric disorders.

## 1 Introduction

Hormones integrate traits into adaptive suites (McGlothlin and Ketterson, 2008). When several traits are regulated by one hormone, their response to evolutionary processes will be linked. Under these circumstances, hormonal mediation may facilitate or restrain phenotypic responses to selection, depending on the form of selection and underlying genetic architecture. Though hormones have adaptive functions, their actions can also predispose individuals to certain conditions, such as cancers and infections (Klein, 2000; Chuffa et al., 2017). Many psychiatric disorders are also linked with variation in hormone expression (Jacobson, 2014; Naughton et al., 2014; Iovino et al., 2018; Gogos et al., 2019). The evolution of hormone-mediated traits involves tradeoffs between their beneficial effects (e.g., maintaining reproduction) and deleterious effects (e.g., susceptibility to cancer).

As an example, estrogen regulates reproductive physiology in women, but a rapid drop in estrogen following parturition leaves women susceptible to depressive episodes (Schiller et al., 2015; Yim et al., 2015). Estrogen’s involvement in both phenotypes provides the basis for a tradeoff between the two. In the presence of tradeoffs, predicting the evolutionary response of traits can be complex and requires a rigorous framework for analysis. The direction of selection on traits involved must be measured empirically because it may differ between traits within the same hormone-mediated suite (McGlothlin and Ketterson, 2008).

Prior work also implicates hormones in the modulation of person perception (Gangestad and Thornhill, 2008; Romero-Martinez et al., 2021). As part of hormone-mediated suites, person perception is unlikely to evolve independently of other traits regulated by the same hormones. In this Perspectives article, we focus on estrogen because it directly influences fitness components and is a well-studied influence on person perception and psychiatric disorders. We introduce evolutionary quantitative genetics to provide a framework for discussion. We then review known and emerging functions of estrogen. Finally, we discuss the evolutionary dynamics of sex hormone-mediated suites and implications for the evolution of psychiatric disorders and person perception. Our hope is that the framework discussed here could similarly be applied to other traits of interest to evolutionary psychology as has been accomplished robustly in behavioral quantitative genetics (Boake, 1994).

### 1.1 Quantitative genetics – a framework for testing evolutionary hypotheses

Darwin’s theory of evolution by natural selection can be distilled to the following syllogism: for a given population, if more offspring are produced than survive to reproduce, and if individuals vary in their traits (including fitness), and if some of that variation is heritable, then that population will evolve (Darwin, 1859). Darwin recognized a second mechanism, sexual selection, which hinges on variation in the ability to obtain mates (via intrasexual competition or mate choice) rather than variation in survival. This theory was formalized beginning in the 20th century by Ronald Fisher and Sewell Wright, marking the birth of evolutionary quantitative genetics (Lynch and Walsh, 1998; Mayr and Provine, 1998). This approach focuses on phenotypic variation associated with multiple genetic loci, which is the case for most behaviors, psychological traits, and psychiatric disorders (Geschwind and Flint, 2015). A deceptively simple set of equations may be used to predict how variation in quantitative traits is expected to respond to natural or sexual selection. Quantitative genetics thus provides an analytically rigorous framework for applying evolutionary theory to and empirically testing hypotheses posited by evolutionary psychology.

First, phenotypic variance can be decomposed into multiple sources. Twin studies are the most commonly used quantitative genetic study design for psychiatric disorders and psychological traits (Kendler, 1993; Kendler, 2001; Vukasovic and Bratko, 2015). Modern quantitative genetics benefits from pedigree-based studies, including the use of the “animal model,” which permits the use of pedigrees that are missing measurement of traits in some individuals and allows for more precise estimation of variance components (Kruuk, 2004; Kruuk and Hadfield, 2007). When combined with adoption studies, common environmental effects can be estimated (Lynch and Walsh, 1998). If pedigrees are sufficiently large and complex (e.g., contain mothers who have offspring from different fathers) or contain repeated measurements, other parameters such as maternal effects and permanent environmental effects may be estimated. The basic equation describing decomposition of phenotypic variance is given as:


P=G+E+G×E+R

In this equation, P refers to phenotypic variance, G to genetic variance, E to environmental variance, GxE to variance from gene x environment effects, and R to residual variance (Falconer and Mackay, 1996). Genetic variance may arise from additive genetic variation, dominance, or epistasis. The proportion of phenotypic variance accounted for by genetic variance is called broad-sense heritability. Sources of environmental variance include parental effects, common environmental effects, or permanent environmental effects. GxE effects occur when different genotypes respond to environmental change in nonparallel ways. For all psychiatric disorders studied, phenotypic variance results from both environmental and genetic sources (Sullivan and Geschwind, 2019). Face perception likewise has both environmental and heritable sources of variance (Zhu et al., 2010).

For predicting the response to selection, two key parameters are important: narrow-sense heritability and the strength of selection (Lush, 1937; Walsh and Lynch, 2018). Narrow-sense heritability (*h*^2^) is defined as the proportion of phenotypic variance (*P*) due to additive genetic effects (*A*) (Falconer and Mackay, 1996; Lynch and Walsh, 1998):


$$h^{2}=\frac{A}{P}$$

Of note, narrow-sense heritability describes only part of the resemblance between relatives’ phenotypes. Other sources of genetic variation will also cause resemblance among relatives. Quantitative genetics focuses on narrow-sense heritability, however, because this component responds to selection. Common environmental and parental effects may also cause resemblance between siblings and can inflate estimates of heritability if not measured (Lynch and Walsh, 1998). Some variance due to epigenetics may be heritable; recent extensions of quantitative genetic models incorporate this (Jablonka and Raz, 2009; Franklin et al., 2010; Stopher et al., 2012; Thomson et al., 2018). In humans, cultural inheritance is especially important and has also recently received attention within quantitative genetics (Danchin et al., 2011, 2013). Any trait with non-zero heritability has the potential to respond to selection.

When examining suites of traits, however, heritability must be extended to account for genetic correlations between those traits. The multivariate extension of heritability is defined by a matrix of additive genetic variances and covariances among traits called the G-matrix. From the G-matrix, one can calculate genetic correlations (r_G_):


$$r_{G}=\frac{cov\;\left(A_{1},A_{2}\right)}{\sqrt{A_{1}\cdot A_{2}}}$$

The genetic correlation is simply the Pearson correlation between the additive genetic components of two traits (*A*_1_ and *A*_2_). Recent studies suggest genetic correlations among many psychiatric disorders (Brainstorm et al., 2018; Grotzinger et al., 2022). Interestingly, another recent study suggested that psychiatric disorders are genetically correlated most strongly with pulmonary, gastrointestinal, and neurological disorders (Athanasiadis et al., 2022). Collectively, these studies imply that evolution of traits involved in one psychiatric disorder will depend on evolution of traits involved not only in other psychiatric disorders, but also in disorders involving organs beyond the brain. Similarly, genetic correlations among scores in the Minnesota Multiphasic Personality Inventory would suggest that personality traits will not evolve entirely independently of each other – a pattern of great import for the evolutionary psychology of personality (Viken and Rose, 2007).

The second key parameter, the strength of selection, was formalized by George Price (Price, 1970, 1972). The “Price Equation” defines selection on a phenotype as the covariance between that phenotype (*P*) and fitness (*ω*):


$$S=cov\left(P,\omega \right)$$

Fitness in evolutionary genetics is defined as differential reproductive success—an individual’s lifetime number of pregnancies relative to the population mean. The average number of children varies widely among human populations (United Nations, 2022). Because usually S < 1, variance in relative reproductive success sets the maximum potential response of a trait to selection, known as the “opportunity for selection” (Crow, 1958). According to Fisher, the strength of selection is more accurately defined by the genetic correlation between a phenotype and fitness, though this is rarely empirically measured (Fisher, 1958). Of note for studies of sexual selection, three covariances are important: between a trait and relative mating success, between a trait and relative reproductive success, and between relative mating success and relative reproductive success (Arnold and Wade, 1984). When there is no relationship between relative mating success and relative reproductive success, no sexual selection can occur. Sexual and natural selection may oppose each other in direction.

A few studies have examined fitness consequences of psychiatric disorders. Bipolar disorder appears to reduce fecundity, yet may be associated with increased fertility at younger ages (Power et al., 2013; Jacobson, 2016; Grover et al., 2019; Hope et al., 2020). Compared to women without psychiatric disorders, affected women are at risk for several negative fitness outcomes, including recurrent miscarriage, sexually transmitted infections, and reproductive cancer (Hope et al., 2022). Higher than average anxiety, on the other hand, was associated with quadratic (U-shaped) increases in fitness with individuals showing lower than average or higher than average anxiety having more children (Jacobson and Roche, 2018). For major depressive disorder, one study showed affected individuals do not have decreased fecundity when compared to their siblings, but another showed that affected individuals have lower fecundity compared with the general population (Tondo et al., 2011; Power et al., 2013). Although the relationship between psychiatric disorders and fitness outcomes has been preliminarily examined, the association between other psychological traits and fitness outcomes warrants further study, as this relationship is key to understanding their contemporary evolution.

The “Breeder’s Equation” describes the expected response to selection (Lush, 1937):


$$R=h^{2}S$$

The response to selection for any given trait (*R*) is that trait’s heritability multiplied by the strength of selection. Whenever *h*^2^ < 1, the effect of selection on a trait is proportionally diminished.

The multivariate extension of the Breeder’s Equation allows for estimation of response of a suite of traits to selection (Lande, 1979). This equation is especially important for hormone-mediated suites of traits:


$$\Delta z^{-}={GP}^{-1}S$$

Here, Δz̄ is the vector of responses in a suite of phenotypes, *G* is the G-matrix, *P*-1 is the phenotypic variance–covariance matrix for the traits, and S is the vector of selection differentials on those traits. The pertinent consequence of this equation for hormone-mediated suites is that the response of one trait to selection depends on selection directly on that trait in addition to selection on every other trait with which it is genetically correlated (Lande and Arnold, 1983). When one trait responds to selection on another trait with which it is genetically correlated, the process is called correlational selection. Within a suite of genetically correlated traits, direct selection on each trait may differ in strength, form, or direction. The overall direction of change for a trait in response to selection, thus, does not only depend on selection directly on that trait, but on correlational selection through other traits as well (Lande and Arnold, 1983; Arnold, 1992). To our knowledge, no studies have used the quantitative genetic framework to predict response to selection for psychiatric disorders or other psychological traits.

Hormonal pleiotropy is an important source of genetic covariance (Wittman et al., 2021). For traits involved in hormone-mediated suites, evolution of each of those traits will potentially be dependent on selection acting on multiple others. This may cause the trait of interest to respond to selection in ways not predicted by univariate models (Arnold, 1992; McGlothlin and Ketterson, 2008).

Some properties of quantitative genetic parameters are largely overlooked, but have important implications for evolutionary psychology. First, these parameters are properties of populations, not individuals. Second, all quantitative genetic parameters are specific to the age during which they are measured and can change over the life of an organism. Prior empirical work demonstrates that heritability and other quantitative genetic parameters can change significantly with age (Wilson et al., 2005). Selection may favor increases in a phenotype in young individuals and decreases in that phenotype in older individuals (Roff, 1992; Stearns, 1992). Third, these parameters are specific to the population in which they are measured. One ought not assume that heritability measured in one population will be the same as heritability measured in another. Finally, quantitative genetic parameters are specific to the generation in which they are measured and may change in response to different processes, including selection (Athanasiadis et al., 2022). Selection may change strength, form, or direction from one generation to the next and in turn alter trait heritability. For these reasons, the adaptive value of a trait in the past or future should be distinguished from current selection on that trait. This discordance is called “mismatch” (Corbett et al., 2018). For example, the neurobiological systems regulating response to reward are not adapted to stimuli from recreational drugs, and those with substance use disorders may have lower lifetime reproductive success (Nesse and Berridge, 1997; Troisi, 2001; Jacobson, 2016). Other examples of quantitative genetic predictions of hypotheses from the evolutionary psychology literature are given in Table 1. These examples illustrate how quantitative genetics may analytically bridge evolutionary theory with evolutionary psychology by allowing for empirical tests of hypotheses about how evolutionary processes shape variation in psychological traits.

**Table 1:** Examples of hypotheses from evolutionary psychology and their concordant quantitative genetic predictions.

Evolutionary psychology hypothesis	Quantitative genetic predictions
Trait A is adaptive	Positive covariance between Trait A and fitness outcomes
Trait B is deleterious	Negative covariance between Trait B and fitness outcomes
Trait C is the result of a historical process of strong directional selection	Low heritability of Trait C
Trait D is an adaptation to environment E, which is deleterious in environment F (“mismatch”)	Positive covariance between Trait D and fitness in environment E, but negative covariance between Trait D and fitness in environment F, e.g., Hereford (2009)
Patterns of personality covariance are adaptive strategies	Genetic integration among personality traits with concordant fitness benefits, e.g., Duckworth and Kruuk (2009)
The mind is organized into adaptive modules	Pattern of genetic covariance between traits within the same posited modules and weak or limited genetic covariance between traits within different posited modules, e.g., Drake and Klingenberg (2010)
Emotion G has a specific function, H	Positive covariance between performance of the posited function and fitness, e.g., Arnold (1983)
Person perception is an adaptation that facilitates mate choice	Covariance between person perception traits and mate choice AND covariance between mate choice and fitness outcomes, e.g., Brooks and Endler (2001)

Empirical work investigating some of these examples in non-human taxa is provided.

### 1.2 The estrogen-mediated suite of traits

Sex hormones are privileged with regard to selection because they regulate reproductive traits and thus generate strong potential for correlational selection among other traits in their suite (McGlothlin and Ketterson, 2008). In humans, sex hormones are well-studied for their effects on the development of sexual characteristics. Here, we focus on estrogen, but the principles we elucidate should be considered for other sex hormones.

**Figure 1:**
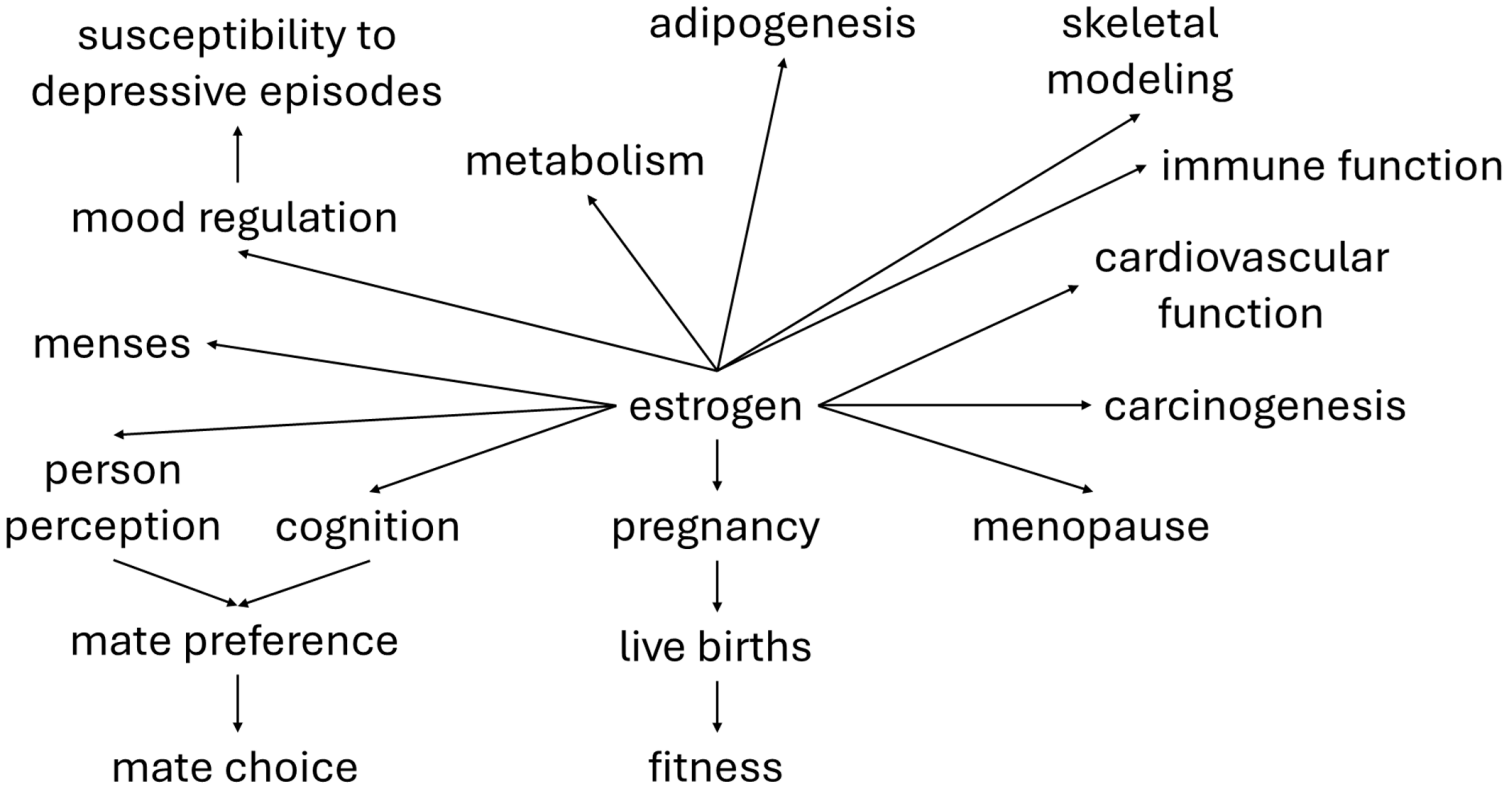
The pleiotropic effects of estrogen. Due to the reproductive effects of estrogen, its correlation with fitness should be quite strong, providing the basis for correlational selection on other traits that it regulates. The strength and direction of genetic correlations between traits within this estrogen-mediated suite partially determines whether the evolutionary response of individual traits is facilitated or constrained. A thorough understanding of the function of hormone-regulated traits must include consideration of this underlying genetic architecture.

Estrogen signaling, in addition to regulating growth, development, and physiology of female reproduction, is crucial for the timing of life history transitions (e.g., menarche and menopause), metabolism, immune function, adipogenesis, skeletal modeling, cardiovascular system functioning, and mood regulation (Dluzen, 2005; Kovats, 2015). Estrogen may also regulate mate preference (Gangestad and Thornhill, 2008; Jünger et al., 2018). Additionally, estrogen is a major factor in carcinogenesis, especially in cancers of the breast and female reproductive tract (Grady et al., 1995; Clemons and Goss, 2001; Kaaks et al., 2002; Rossouw et al., 2002; Yager and Davidson, 2006; Reid et al., 2017). This network of traits influenced by estrogen provides the architecture for correlational selection (Figure 1).

Estrogen signaling accomplishes this diversity of functions by acting through several molecular mechanisms, including binding to receptors or directly to DNA to alter transcription (Bjornstrom and Sjoberg, 2005). Serum estradiol concentration is regulated by sex hormone binding globulin (Siiteri et al., 1982; Arathimos et al., 2020). Free estradiol may be important for some phenotypes, such as carcinogenesis, while fluctuations in hormone levels are important for others, such as menstrual cycles, maintenance and progression of pregnancies, and mood regulation (Deroo and Korach, 2006; Burns and Korach, 2012; Christensen et al., 2012; Hamilton et al., 2017; Gordon et al., 2019). Nuances in the mechanisms by which a suite of traits is regulated allow for partial independence in the evolution of those traits when r_G_ < 1 (Wagner and Lynch, 2008).

Though some of the functions of estrogen are limited to women, estrogen is important in regulating several functions in men as well (Kousteni et al., 2001). This scenario is the basis for cross-sex genetic correlations (r_MF_), whereby evolution of trait expression in men will be linked to the evolution of those same traits in women. Cross-sex genetic correlations have been the target of intensive work in evolutionary biology due to the role that this genetic architecture can play in sexual selection (Badyaev, 2002). The major consequence of r_MF_ is that selection on one sex will generate selection in the other. If r_MF_ between the sexes is negative, and selection on the sexes is in opposite directions, the evolution of sex differences is expected (Lande, 1980). On the other hand, if r_MF_ is positive and selection on the sexes is in opposite directions, the evolution of sex differences will be constrained. The long-term outcomes of sex differences depend on the strength, direction, and consistency of selection and the strength and direction of r_MF_ (Badyaev, 2002; Walsh and Lynch, 2018).

Estrogen is implicated in several psychiatric disorders and may be partially responsible for observed sex differences. Sex differences in risk of depression, for example, emerge in puberty and continue throughout life (Kessler, 2003). This risk is mechanistically linked with fluctuations in estrogen levels, for example during pregnancy, peripartum periods, and menopause (Payne, 2003; Bennett et al., 2004; Freeman et al., 2006; O'Hara and Swain, 2009; O'Hara and McCabe, 2013). Estrogen, by regulating mood, is associated with both major depressive disorder and bipolar disorder (Halbreich and Kahn, 2001; Borrow and Cameron, 2014; Frey and Dias, 2014). Cycle-related changes in mood symptoms in both these disorders have also been reported (Payne et al., 2007). Estrogen and progesterone jointly increase vulnerability for developing anxiety disorders and influence the presentation, course, and treatment response of anxiety disorders, especially in women (Pigott et al., 2019). In schizophrenia, several possible mechanisms also associate estrogen signaling with cognitive function (McGregor et al., 2017). Estrogen receptor expression in the frontal cortex and hippocampus shows sex differences in major depressive disorder, but not in schizophrenia and bipolar disorder (Perlman et al., 2005). Sex differences in susceptibility to neurodegenerative diseases may also be related to neuroprotective effects of estrogen (Vegeto et al., 2020).

## 2 Discussion

When an array of phenotypes is regulated by one hormone, predicting the response to selection of a single phenotype is difficult without large sampling and detailed phenotyping efforts. Within the field of hormones and person perception, most efforts have focused on relating estrogen function individually to various phenotypes, such as mate choice or preference (Feinberg et al., 2006; Garver-Apgar et al., 2008; Lukaszewski and Roney, 2009). Large quantitative genetic studies within these fields could provide important empirical tests of theory, especially considering existing methods to construct pedigrees from already collected genetic data (Staples et al., 2014). For clinical fields, evolutionary theory can shed light on unresolved fundamental questions about the nature of psychiatric disorders (Nesse, 2023).

Hormonal regulation of person perception is a key area for understanding how psychiatric disorders evolve due to its role in sexual selection. Sex hormones are associated with variation in perception of faces, morphology, and emotions (Gangestad and Thornhill, 2008; Romero-Martinez et al., 2021). Person perception varies between populations and biological sexes, is influenced by psychiatric disorders, and changes with reproductive physiology, season, and age (Gangestad and Thornhill, 2008; Pawlowski and Sorokowski, 2008; Kohler et al., 2010, 2011; Boothroyd and Vukovic, 2019; Olderbak et al., 2019). This abundant phenotypic variation suggests ample opportunity for evolutionary processes to change the distribution of these traits from one generation to the next.

Crucially, hormonal regulation of person perception modifies the sensory processes necessary for expressing mating preference and choice, but is not equivalent to these. Mating preference and mate choice must be measured empirically, a problem which presents unique methodological challenges (Andersson and Simmons, 2006; Dougherty, 2020; Clancey et al., 2022). Variation in the perception of mates over time (e.g., across the menstrual cycle) allows for changes in mate preference, but these changes do not necessarily entail changes in mate choice, especially given variation in human mating systems (Todd et al., 2007; Schacht and Kramer, 2019). In turn, changes variation in mate choice may or may not be under natural or sexual selection depending on the relationship between mate choice and fitness (Shuster and Wade, 2003; Walsh and Lynch, 2018). For example, in many human societies, marriage is primarily an economic transaction involving the influence of an individual’s parents or family (Ingoldsby, 2006; Buunk et al., 2009). In these societies, the expression of individual preference may be facilitated or opposed by familial influence. The fitness consequences (e.g., frequency of extrapair mating) can depend on alignment of individual and familial preferences (Scelza, 2011). Quantitative genetics provides a robust framework for studying the role of hormonal regulation of person perception in sexual selection as well as the evolution of psychiatric disorders in the setting of hormone-mediated suites of traits.

More empirical work is needed to clarify several evolutionary quantitative genetic questions about hormones and person perception, such as quantification of univariate and multivariate heritability of hormone-mediated suites. For reasons outlined above, this work must include studies of different populations, ages, and generations. Most studies of heritability of clinical phenotypes do not examine these differences. The unwarranted, tacit assumption is that risky genetic loci are stable across these contexts or change on longer timescales than are clinically relevant. Given that sex-hormone mediated traits undergo large regulatory changes during puberty, these knowledge gaps are especially important targets for empirical work. Few studies have quantified contemporary selection on hormones and person perception or examined mechanisms of selection [though see (Hill et al., 2013)], which means the processes possibly driving changes in heritability are almost completely overlooked.

Overall, we have discussed beneficial and adverse consequences of sex hormone signaling, highlighting the fact that tradeoffs among various functions of hormonal suites are common. This discussion emphasizes the importance of caution in drawing conclusions about the evolution of psychological traits and psychiatric disorders from single phenotype studies. This caveat is especially important for the field of hormones and person perception due to the possibility of conflicts between sexual and natural selection. Our hope is that future work will incorporate evolutionary quantitative genetic approaches to studying adaptive hormone-mediated suites of traits.

## Data availability statement

The original contributions presented in the study are included in the article/supplementary material, further inquiries can be directed to the corresponding author.

## Author contributions

CG: Writing – review & editing, Writing – original draft, Visualization, Conceptualization. TK: Writing – review & editing, Supervision, Conceptualization. TP: Writing – review & editing, Supervision, Conceptualization.

## References

[ref1] Andersson M. Simmons L. W. (2006). Sexual selection and mate choice. Trends Ecol. Evol. 21, 296–302. doi: 10.1016/j.tree.2006.03.015 16769428

[ref2] Arathimos R. Millard L. A. C. Bell J. A. Relton C. L. Suderman M. (2020). Impact of sex hormone-binding globulin on the human phenome. Hum. Mol. Genet. 29, 1824–1832. doi: 10.1093/hmg/ddz269, PMID: 32533189 PMC7372548

[ref3] Arnold S. J. (1983). Morphology, performance and fitness. Am. Zool. 23, 347–361. doi: 10.1093/icb/23.2.347

[ref4] Arnold S. J. (1992). Constraints on phenotypic evolution. Am. Nat. 140, S85–S107. doi: 10.1086/285398 19426028

[ref5] Arnold S. J. Wade M. J. (1984). On the measurement of natural and sexual selection: theory. Evolution 38, 709–719. doi: 10.2307/2408383 28555816

[ref6] Athanasiadis G. Meijsen J. J. Helenius D. Schork A. J. Ingason A. Thompson W. K.. (2022). A comprehensive map of genetic relationships among diagnostic categories based on 48.6 million relative pairs from the Danish genealogy. Proc. Natl. Acad. Sci. USA 119, 1–9. 10.1073/pnas.2118688119 PMC8833149 35131856

[ref7] Badyaev A. V. (2002). Growing apart: an ontogenetic perspective on the evolution of sexual size dimorphism. Trends Ecol. Evol. 17, 369–378. doi: 10.1016/S0169-5347(02)02569-7

[ref8] Bennett H. A. Einarson A. Taddio A. Koren G. Einarson T. R. (2004). Prevalence of depression during pregnancy: systematic review. Obstet. Gynecol. 103, 698–709. doi: 10.1097/01.AOG.0000116689.75396.5f 15051562

[ref9] Bjornstrom L. Sjoberg M. (2005). Mechanisms of estrogen receptor signaling: convergence of genomic and nongenomic actions on target genes. Mol. Endocrinol. 19, 833–842. doi: 10.1210/me.2004-0486, PMID: 15695368

[ref10] Boake C. R. B. (1994). Quantitative Genetic Studies of Behavioral Evolution. Chicago: University of Chicago Press.

[ref11] Boothroyd L. G. Vukovic J. (2019). “Mate preferences across the lifespan” in The Oxford handbook of evolutionary psychology and behavioral endocrinology. eds. Welling L. L. M. Shackelford T. K. (New York, NY, USA: Oxford University Press).

[ref12] Borrow A. P. Cameron N. M. (2014). Estrogenic mediation of serotonergic and neurotrophic systems: implications for female mood disorders. Prog. Neuro-Psychopharmacol. Biol. Psychiatry 54, 13–25. doi: 10.1016/j.pnpbp.2014.05.009, PMID: 24865152

[ref13] Brainstorm C. Anttila V. Bulik-Sullivan B. Finucane H. K. Walters R. K. Bras J.. (2018). Analysis of shared heritability in common disorders of the brain. Science 360, 1–12. 10.1126/science.aap8757 PMC6097237 29930110

[ref14] Brooks R. Endler J. A. (2001). Female guppies agree to differ: phenotypic and genetic variation in mate-choice behavior and the consequences for sexual selection. Evolution 55, 1644–1655, PMID: 11580024 10.1111/j.0014-3820.2001.tb00684.x

[ref15] Burns K. A. Korach K. S. (2012). Estrogen receptors and human disease: an update. Arch. Toxicol. 86, 1491–1504. doi: 10.1007/s00204-012-0868-5, PMID: 22648069 PMC4782145

[ref16] Buunk A. P. Park J. H. Duncan L. A. (2009). Cultural variation in parental influence on mate choice. Cross-Cult. Res. 44, 23–40.

[ref17] Christensen A. Bentley G. E. Cabrera R. Ortega H. H. Perfito N. Wu T. J.. (2012). Hormone regulation of female reproduction. Horm. Metab. Res. 44, 587–591. doi: 10.1055/s-0032-1306301, PMID: 22438212 PMC3647363

[ref18] Chuffa L. G. Lupi-Junior L. A. Costa A. B. Amorim J. P. Seiva F. R. (2017). The role of sex hormones and steroid receptors on female reproductive cancers. Steroids 118, 93–108. doi: 10.1016/j.steroids.2016.12.011 28041951

[ref19] Clancey E. Johnson T. R. Harmon L. J. Hohenlohe P. A. (2022). Estimation of the strength of mate preference from mated pairs observed in the wild. Evolution 76, 29–41. doi: 10.1111/evo.14397, PMID: 34792183 PMC9300214

[ref20] Clemons M. Goss P. (2001). Estrogen and the risk of breast cancer. N. Engl. J. Med. 344, 276–285. doi: 10.1056/NEJM200101253440407 11172156

[ref21] Corbett S. Courtiol A. Lummaa V. Moorad J. Stearns S. (2018). The transition to modernity and chronic disease: mismatch and natural selection. Nat. Rev. Genet. 19, 419–430. doi: 10.1038/s41576-018-0012-3, PMID: 29743650

[ref22] Crow J. F. (1958). Some possibilities for measuring selection intensities in man. Hum. Biol. 61, 763–775. 2699600

[ref23] Danchin E. Charmantier A. Champagne F. A. Mesoudi A. Pujol B. Blanchet S. (2011). Beyond DNA: integrating inclusive inheritance into an extended theory of evolution. Nat. Rev. Genet. 12, 475–486. doi: 10.1038/nrg3028, PMID: 21681209

[ref24] Danchin E. Pujol B. Wagner R. H. (2013). The double pedigree: a method for studying culturally and genetically inherited behavior in tandem. PLoS One 8:e61254. doi: 10.1371/journal.pone.0061254 23700404 PMC3659024

[ref25] Darwin C. (1859). On the origin of species by means of natural selection or the preservation of favoured races in the struggle for life. Edison, NJ, USA: Castle Books. PMC5184128 30164232

[ref26] Deroo B. J. Korach K. S. (2006). Estrogen receptors and human disease. J. Clin. Invest. 116, 561–570. doi: 10.1172/JCI27987 16511588 PMC2373424

[ref27] Dluzen D. E. (2005). Unconventional effects of estrogen uncovered. Trends Pharmacol. Sci. 26, 485–487. doi: 10.1016/j.tips.2005.08.001, PMID: 16122814

[ref28] Dougherty L. R. (2020). Designing mate choice experiments. Biol. Rev. Camb. Philos. Soc. 95, 759–781. doi: 10.1111/brv.12586, PMID: 32022418

[ref29] Drake A. G. Klingenberg C. P. (2010). Large-scale diversification of skull shape in domestic dogs: disparity and modularity. Am. Nat. 175, 289–301. doi: 10.1086/650372, PMID: 20095825

[ref30] Duckworth R. A. Kruuk L. E. (2009). Evolution of genetic integration between dispersal and colonization ability in a bird. Evolution 63, 968–977. doi: 10.1111/j.1558-5646.2009.00625.x 19154391

[ref31] Falconer D. S. Mackay T. F. C. (1996). Introduction to quantitative genetics. Essex, U.K.: Longman.

[ref32] Feinberg D. R. Jones B. C. Law Smith M. J. Moore F. R. DeBruine L. M. Cornwell R. E.. (2006). Menstrual cycle, trait estrogen level, and masculinity preferences in the human voice. Horm. Behav. 49, 215–222. doi: 10.1016/j.yhbeh.2005.07.004, PMID: 16055126

[ref33] Fisher R. A. (1958). The Genetical theory of natural selection. Toronto: Dover.

[ref34] Franklin T. B. Russig H. Weiss I. C. Graff J. Linder N. Michalon A.. (2010). Epigenetic transmission of the impact of early stress across generations. Biol. Psychiatry 68, 408–415. doi: 10.1016/j.biopsych.2010.05.036 20673872

[ref35] Freeman E. W. Sammel M. D. Lin H. Nelson D. B. (2006). Associations of hormones and menopausal status with depressed mood in women with no history of depression. Arch. Gen. Psychiatry 63, 375–382. doi: 10.1001/archpsyc.63.4.375 16585466

[ref36] Frey B. N. Dias R. S. (2014). Sex hormones and biomarkers of neuroprotection and neurodegeneration: implications for female reproductive events in bipolar disorder. Bipolar Disord. 16, 48–57. doi: 10.1111/bdi.12151, PMID: 24206266

[ref37] Gangestad S. W. Thornhill R. (2008). Human oestrus. Proc. Biol. Sci. 275, 991–1000. 18252670 10.1098/rspb.2007.1425 PMC2394562

[ref38] Garver-Apgar C. E. Gangestad S. W. Thornhill R. (2008). Hormonal correlates of women's mid-cycle preference for the scent of symmetry. Evol. Hum. Behav. 29, 223–232. doi: 10.1016/j.evolhumbehav.2007.12.007

[ref39] Geschwind D. H. Flint J. (2015). Genetics and genomics of psychiatric disease. Science 349, 1489–1494. doi: 10.1126/science.aaa8954, PMID: 26404826 PMC4694563

[ref40] Gogos A. Ney L. J. Seymour N. Van Rheenen T. E. Felmingham K. L. (2019). Sex differences in schizophrenia, bipolar disorder, and post-traumatic stress disorder: are gonadal hormones the link? Br. J. Pharmacol. 176, 4119–4135. doi: 10.1111/bph.14584, PMID: 30658014 PMC6877792

[ref41] Gordon J. L. Peltier A. Grummisch J. A. Sykes Tottenham L. (2019). Estradiol fluctuation, sensitivity to stress, and depressive symptoms in the menopause transition: a pilot study. Front. Psychol. 10:1319. doi: 10.3389/fpsyg.2019.01319, PMID: 31244722 PMC6581734

[ref42] Grady D. Gebretsadik T. Kerlikowske K. Ernster V. Petitti D. (1995). Hormone replacement therapy and endometrial cancer risk: a Meta-analysis. Obstet. Gynecol. 85, 304–313. doi: 10.1016/0029-7844(94)00383-O, PMID: 7824251

[ref43] Grotzinger A. D. Mallard T. T. Akingbuwa W. A. Ip H. F. Adams M. J. Lewis C. M.. (2022). Genetic architecture of 11 major psychiatric disorders at biobehavioral, functional genomic and molecular genetic levels of analysis. Nat. Genet. 54, 548–559. 35513722 10.1038/s41588-022-01057-4 PMC9117465

[ref44] Grover S. Sahoo S. Chaudhary S. Chakrabarti S. Nehra R. Avasthi A. (2019). Gender differences, family size and fertility rate among patients with bipolar disorder: a study from India. Psychiatry Res. 272, 562–568. doi: 10.1016/j.psychres.2018.12.156, PMID: 30616124

[ref45] Halbreich U. Kahn L. S. (2001). Role of estrogen in the Aetiology and treatment of mood disorders. CNS Drugs 15, 797–817. doi: 10.2165/00023210-200115100-00005 11602005

[ref46] Hamilton K. J. Hewitt S. C. Arao Y. Korach K. S. (2017). Estrogen Hormone Biology. Curr. Top. Dev. Biol. 125, 109–146. doi: 10.1016/bs.ctdb.2016.12.005 28527569 PMC6206851

[ref47] Hereford J. (2009). A quantitative survey of local adaptation and fitness trade-offs. Am. Nat. 173, 579–588. doi: 10.1086/597611, PMID: 19272016

[ref48] Hill A. K. Hunt J. Welling L. L. M. Cárdenas R. A. Rotella M. A. Wheatley J. R.. (2013). Quantifying the strength and form of sexual selection on men's traits. Evol. Hum. Behav. 34, 334–341. doi: 10.1016/j.evolhumbehav.2013.05.004

[ref49] Hope H. Parisi R. Ashcroft D. M. Williams R. Coton S. Kosidou K.. (2020). Fertility trends of women with serious mental illness in the United Kingdom 1992-2017: a primary care cohort study using the clinical practice research datalink. J. Affect. Disord. 269, 141–147. doi: 10.1016/j.jad.2020.03.037, PMID: 32250867

[ref50] Hope H. Pierce M. Johnstone E. D. Myers J. Abel K. M. (2022). The sexual and reproductive health of women with mental illness: a primary care registry study. Arch. Womens Ment. Health 25, 585–593. doi: 10.1007/s00737-022-01214-y, PMID: 35366692 PMC9072520

[ref51] Ingoldsby B. B. (2006). “Mate selection and marriage” in Families in global and multicultural perspective. eds. Ingoldsby B. B. Smith S. D. (Thousand Oaks, CA, USA: Sage Publications).

[ref52] Iovino M. Messana T. De Pergola G. Iovino E. Dicuonzo F. Guastamacchia E.. (2018). The role of Neurohypophyseal hormones vasopressin and oxytocin in neuropsychiatric disorders. Endocr. Metab. Immune Disord. Drug Targets 18, 341–347. doi: 10.2174/1871530318666180220104900, PMID: 29468985

[ref53] Jablonka E. Raz G. (2009). Transgenerational epigenetic inheritance: prevalence, mechanisms, and implications for the study of heredity and evolution. Q. Rev. Biol. 84, 131–176. doi: 10.1086/598822, PMID: 19606595

[ref54] Jacobson L. (2014). Hypothalamic-pituitary-adrenocortical axis: neuropsychiatric aspects. Compr. Physiol. 4, 715–738. doi: 10.1002/cphy.c130036, PMID: 24715565

[ref55] Jacobson N. C. (2016). Current evolutionary adaptiveness of psychiatric disorders: fertility rates, parent-child relationship quality, and psychiatric disorders across the lifespan. J. Abnorm. Psychol. 125, 824–839. doi: 10.1037/abn0000185, PMID: 27362490 PMC4980185

[ref56] Jacobson N. C. Roche M. J. (2018). Current evolutionary adaptiveness of anxiety: extreme phenotypes of anxiety predict increased fertility across multiple generations. J. Psychiatr. Res. 106, 82–90. doi: 10.1016/j.jpsychires.2018.10.002, PMID: 30296705 PMC6219631

[ref57] Jünger J. Kordsmeyer T. L. Gerlach T. M. Penke L. (2018). Fertile women evaluate male bodies as more attractive, regardless of masculinity. Evol. Hum. Behav. 39, 412–423. doi: 10.1016/j.evolhumbehav.2018.03.007

[ref58] Kaaks R. Lukanova A. Kurzer M. S. (2002). Obesity, endogenous hormones, and endometrial cancer risk: a synthetic review. Am. Soc. Prevent. Oncol. 11, 1531–1543, PMID: 12496040

[ref59] Kendler K. S. (1993). Twin studies of psychiatric illness: current status and future directions. Arch. Gen. Psychiatry 50, 905–915. doi: 10.1001/archpsyc.1993.01820230075007 8215816

[ref60] Kendler K. S. (2001). Twin studies of psychiatric illness: an update. Arch. Gen. Psychiatry 58, 1005–1014. doi: 10.1001/archpsyc.58.11.1005 11695946

[ref61] Kessler R. (2003). Epidemiology of women and depression. J. Affect. Disord. 74, 5–13. doi: 10.1016/S0165-0327(02)00426-3 12646294

[ref62] Klein S. L. (2000). The effects of hormones on sex differences in infection: from genes to behavior. Neurosci. Biobehav. Rev. 24, 627–638. doi: 10.1016/S0149-7634(00)00027-0, PMID: 10940438

[ref63] Kohler C. G. Hoffman L. J. Eastman L. B. Healey K. Moberg P. J. (2011). Facial emotion perception in depression and bipolar disorder: a quantitative review. Psychiatry Res. 188, 303–309. doi: 10.1016/j.psychres.2011.04.019 21601927

[ref64] Kohler C. G. Walker J. B. Martin E. A. Healey K. M. Moberg P. J. (2010). Facial emotion perception in schizophrenia: a meta-analytic review. Schizophr. Bull. 36, 1009–1019. doi: 10.1093/schbul/sbn192, PMID: 19329561 PMC2930336

[ref65] Kousteni S. Bellido T. Plotkin L. I. O'Brien C. A. Bodenner D. L. Han L.. (2001). Nongenotropic, sex-nonspecific signaling through the estrogen or androgen receptors: dissociation from transcriptional activity. Cell 104, 719–730, PMID: 11257226

[ref66] Kovats S. (2015). Estrogen receptors regulate innate immune cells and signaling pathways. Cell. Immunol. 294, 63–69. doi: 10.1016/j.cellimm.2015.01.018, PMID: 25682174 PMC4380804

[ref67] Kruuk L. E. (2004). Estimating genetic parameters in natural populations using the "animal model". Philos. Trans. R. Soc. Lond. Ser. B Biol. Sci. 359, 873–890. doi: 10.1098/rstb.2003.1437 15306404 PMC1693385

[ref68] Kruuk L. E. Hadfield J. D. (2007). How to separate genetic and environmental causes of similarity between relatives. J. Evol. Biol. 20, 1890–1903. doi: 10.1111/j.1420-9101.2007.01377.x 17714306

[ref69] Lande R. (1979). Quantitative genetic analysis of multivariate evolution, applied to brain: body size allometry. Evolution 33, 402–416. doi: 10.1111/j.1558-5646.1979.tb04694.x, PMID: 28568194

[ref70] Lande R. (1980). Sexual dimorphism, sexual selection and adaptation in polygenic characters. Evolution 34, 292–305. doi: 10.1111/j.1558-5646.1980.tb04817.x, PMID: 28563426

[ref71] Lande R. Arnold S. J. (1983). The measurement of selection on correlated characters. Evolution 37, 1210–1226. doi: 10.2307/2408842 28556011

[ref72] Lukaszewski A. W. Roney J. R. (2009). Estimated hormones predict women’s mate preferences for dominant personality traits. Personal. Individ. Differ. 47, 191–196. doi: 10.1016/j.paid.2009.02.019

[ref73] Lush J. L. (1937). Animal breeding plans. Ames, IA, USA: Iowa State Press.

[ref74] Lynch M. Walsh B. (1998). Genetics and analysis of quantitative traits. Sunderland, MA, USA: Sinauer Associates, Inc.

[ref75] Mayr E. Provine W. (1998). The evolutionary synthesis: Perspectives on the unification of biology. Cambridge, MA, USA: Harvard University Press.

[ref76] McGlothlin J. W. Ketterson E. D. (2008). Hormone-mediated suites as adaptations and evolutionary constraints. Philos. Trans. R. Soc. Lond. Ser. B Biol. Sci. 363, 1611–1620. doi: 10.1098/rstb.2007.0002, PMID: 18048296 PMC2606720

[ref77] McGregor C. Riordan A. Thornton J. (2017). Estrogens and the cognitive symptoms of schizophrenia: possible neuroprotective mechanisms. Front. Neuroendocrinol. 47, 19–33. doi: 10.1016/j.yfrne.2017.06.003, PMID: 28673758

[ref78] Naughton M. Dinan T. G. Scott L. V. (2014). Corticotropin-releasing hormone and the hypothalamic-pituitary-adrenal axis in psychiatric disease. Handb. Clin. Neurol. 124, 69–91. doi: 10.1016/B978-0-444-59602-4.00005-8 25248580

[ref79] Nesse R. M. (2023). Evolutionary psychiatry: foundations, progress and challenges. World Psychiatry 22, 177–202. doi: 10.1002/wps.21072, PMID: 37159362 PMC10168175

[ref80] Nesse R. M. Berridge K. C. (1997). Psychoactive drug use in evolutionary perspective. Science 278, 63–66. doi: 10.1126/science.278.5335.63 9311928

[ref81] O'Hara M. W. McCabe J. E. (2013). Postpartum depression: current status and future directions. Annu. Rev. Clin. Psychol. 9, 379–407. doi: 10.1146/annurev-clinpsy-050212-185612, PMID: 23394227

[ref82] O'Hara M. W. Swain A. M. (2009). Rates and risk of postpartum depression—a meta-analysis. Int. Rev. Psychiatry 8, 37–54.

[ref83] Olderbak S. Wilhelm O. Hildebrandt A. Quoidbach J. (2019). Sex differences in facial emotion perception ability across the lifespan. Cogn. Emot. 33, 579–588. doi: 10.1080/02699931.2018.1454403, PMID: 29564958

[ref84] Pawlowski B. Sorokowski P. (2008). Men's attraction to women's bodies changes seasonally. Perception 37, 1079–1085. doi: 10.1068/p5715, PMID: 18773730

[ref85] Payne J. L. (2003). The role of estrogen in mood disorders in women. Int. Rev. Psychiatry 15, 280–290. doi: 10.1080/0954026031000136893 15276966

[ref86] Payne J. L. Roy P. S. Murphy-Eberenz K. Weismann M. M. Swartz K. L. McInnis M. G.. (2007). Reproductive cycle-associated mood symptoms in women with major depression and bipolar disorder. J. Affect. Disord. 99, 221–229. doi: 10.1016/j.jad.2006.08.013, PMID: 17011632

[ref87] Perlman W. R. Tomaskovic-Crook E. Montague D. M. Webster M. J. Rubinow D. R. Kleinman J. E.. (2005). Alteration in estrogen receptor alpha mRNA levels in frontal cortex and hippocampus of patients with major mental illness. Biol. Psychiatry 58, 812–824. doi: 10.1016/j.biopsych.2005.04.047, PMID: 16112656

[ref88] Pigott T. A. Duran A. N. Jalnapurkar I. Kimm T. Linscheid S. Allen M. K. (2019). “Sex differences in anxiety disorders” in The Oxford handbook of evolutionary psychology and behavioral endocrinology. eds. Welling L. L. M. Shackelford T. K. (New York, NY, USA: Oxford University Press).

[ref89] Power R. A. Kyaga S. Uher R. MacCabe J. H. Langstrom N. Landen M.. (2013). Fecundity of patients with schizophrenia, autism, bipolar disorder, depression, anorexia nervosa, or substance abuse vs their unaffected siblings. JAMA Psychiatry 70, 22–30. doi: 10.1001/jamapsychiatry.2013.268, PMID: 23147713

[ref90] Price G. R. (1970). Selection and covariance. Nature 227, 520–521. doi: 10.1038/227520a0 5428476

[ref91] Price G. R. (1972). Extension of covariance selection mathematics. Ann. Hum. Genet. 35, 485–490. doi: 10.1111/j.1469-1809.1957.tb01874.x, PMID: 5073694

[ref92] Reid B. M. Permuth J. B. Sellers T. A. (2017). Epidemiology of ovarian cancer: a review. Cancer Biol. Med. 14, 9–32. doi: 10.20892/j.issn.2095-3941.2016.0084, PMID: 28443200 PMC5365187

[ref93] Roff D. A. (1992). Life history evolution. Sinauer, MA, USA: Sinauer Associates, Inc.

[ref94] Romero-Martinez A. Sarrate-Costa C. Moya-Albiol L. (2021). A systematic review of the role of oxytocin, cortisol, and testosterone in facial emotional processing. Biology (Basel) 10, 1–34. doi: 10.3390/biology10121334 PMC8698823 34943249

[ref95] Rossouw J. E. Anderson G. L. Prentice R. L. (2002). Investing in healthy postmenopausal women: principal results from the Women’s health initiative randomized controlled trial. JAMA 288, 321–333. 12117397 10.1001/jama.288.3.321

[ref96] Scelza B. A. (2011). Female choice and extra-pair paternity in a traditional human population. Biol. Lett. 7, 889–891. doi: 10.1098/rsbl.2011.0478, PMID: 21733870 PMC3210680

[ref97] Schacht R. Kramer K. L. (2019). Are we monogamous? A review of the evolution of pair-bonding in humans and its contemporary variation cross-culturally. Front. Ecol. Evol. 7:230. doi: 10.3389/fevo.2019.00230

[ref98] Schiller C. E. Meltzer-Brody S. Rubinow D. R. (2015). The role of reproductive hormones in postpartum depression. CNS Spectr. 20, 48–59. doi: 10.1017/S1092852914000480, PMID: 25263255 PMC4363269

[ref99] Shuster S. M. Wade M. J. (2003). Mating systems and strategies. Princeton, NJ, USA: Princeton University Press.

[ref100] Siiteri P. K. Murai J. T. Hammond G. L. Nisker J. A. Raymoure W. J. Kuhn R. W. (1982). The serum transport of steroid hormones. Recent Prog. Horm. Res. 38, 457–510, PMID: 6750727 10.1016/b978-0-12-571138-8.50016-0

[ref101] Staples J. Qiao D. Cho M. H. Silverman E. K. G. University of Washington Center for Mendelian, D.A. Nickerson, and J.E. Below (2014). PRIMUS: rapid reconstruction of pedigrees from genome-wide estimates of identity by descent. Am. J. Hum. Genet. 95, 553–564. 25439724 10.1016/j.ajhg.2014.10.005 PMC4225580

[ref102] Stearns S. C. (1992). The evolution of life histories. New York, NY, USA: Oxford University Press.

[ref103] Stopher K. V. Walling C. A. Morris A. Guinness F. E. Clutton-Brock T. H. Pemberton J. M.. (2012). Shared spatial effects on quantitative genetic parameters: accounting for spatial autocorrelation and home range overlap reduces estimates of heritability in wild red deer. Evolution 66, 2411–2426. doi: 10.1111/j.1558-5646.2012.01620.x, PMID: 22834741 PMC3437482

[ref104] Sullivan P. F. Geschwind D. H. (2019). Defining the genetic, genomic, cellular, and diagnostic architectures of psychiatric disorders. Cell 177, 162–183. doi: 10.1016/j.cell.2019.01.015, PMID: 30901538 PMC6432948

[ref105] Thomson C. E. Winney I. S. Salles O. C. Pujol B. (2018). A guide to using a multiple-matrix animal model to disentangle genetic and nongenetic causes of phenotypic variance. PLoS One 13:e0197720. doi: 10.1371/journal.pone.0197720, PMID: 30312317 PMC6193571

[ref106] Todd P. M. Penke L. Fasolo B. Lenton A. P. (2007). Different cognitive processes underlie human mate choices and mate preferences. Proc. Natl. Acad. Sci. USA 10, 15011–15016. 10.1073/pnas.0705290104 PMC1986604 17827279

[ref107] Tondo L. Lepri B. Baldessarini R. J. (2011). Reproduction among 1975 Sardinian women and men diagnosed with major mood disorders. Acta Psychiatr. Scand. 123, 283–289. 21219264 10.1111/j.1600-0447.2010.01660.x

[ref108] Troisi A. (2001). Harmful effects of substance abuse: a Darwinian perspective. Funct. Neurol. 16, 237–243, PMID: 11996520

[ref109] United Nations (2022). Department of economic and social affairs, world population prospects 2022: Summary of results. New York, United Nations Publication: UN DESA.

[ref110] Vegeto E. Villa A. Della Torre S. Crippa V. Rusmini P. Cristofani R.. (2020). The role of sex and sex hormones in neurodegenerative diseases. Endocr. Rev. 41, 273–319. doi: 10.1210/endrev/bnz005 31544208 PMC7156855

[ref111] Viken R. J. Rose R. J. (2007). Genetic variation and covariation in the original and restructured clinical scales of the MMPI. J. Abnorm. Psychol. 116, 842–847. doi: 10.1037/0021-843X.116.4.842, PMID: 18020730

[ref112] Vukasovic T. Bratko D. (2015). Heritability of personality: a meta-analysis of behavior genetic studies. Psychol. Bull. 141, 769–785. doi: 10.1037/bul0000017 25961374

[ref113] Wagner G. P. Lynch V. J. (2008). The gene regulatory logic of transcription factor evolution. Trends Ecol. Evol. 23, 377–385. doi: 10.1016/j.tree.2008.03.006 18501470

[ref114] Walsh B. Lynch M. (2018). Evolution and selection of quantitative traits. New York: Oxford University Press.

[ref115] Wilson A. J. Kruuk L. E. Coltman D. W. (2005). Ontogenetic patterns in heritable variation for Body size: using random regression models in a wild ungulate population. Am. Nat. 166, e177–e192. doi: 10.1086/497441, PMID: 16475080

[ref116] Wittman T. N. Robinson C. D. McGlothlin J. W. Cox R. M. (2021). Hormonal pleiotropy structures genetic covariance. Evol Lett 5, 397–407. doi: 10.1002/evl3.240, PMID: 34367664 PMC8327939

[ref117] Yager J. D. Davidson N. E. (2006). Estrogen carcinogenesis in breast Cancer. N. Engl. J. Med. 354, 270–282. doi: 10.1056/NEJMra050776 16421368

[ref118] Yim I. S. Tanner Stapleton L. R. Guardino C. M. Hahn-Holbrook J. Dunkel Schetter C. (2015). Biological and psychosocial predictors of postpartum depression: systematic review and call for integration. Annu. Rev. Clin. Psychol. 11, 99–137. doi: 10.1146/annurev-clinpsy-101414-020426, PMID: 25822344 PMC5659274

[ref119] Zhu Q. Song Y. Hu S. Li X. Tian M. Zhen Z.. (2010). Heritability of the specific cognitive ability of face perception. Curr. Biol. 20, 137–142. doi: 10.1016/j.cub.2009.11.067, PMID: 20060296

